# Study of the Evolution of Pigments from Freshly Pressed to ‘On-the-Shelf’ Extra-Virgin Olive Oils by Means of Near-UV Visible Spectroscopy

**DOI:** 10.3390/foods10081891

**Published:** 2021-08-15

**Authors:** Eleonora Borello, Daniele Roncucci, Valentina Domenici

**Affiliations:** 1 Dipartimento di Chimica e Chimica Industriale, Università di Pisa, Via Moruzzi 13, 56124 Pisa, Italy; bore.89.ele@gmail.com (E.B.); daniele.roncucci@empa.ch (D.R.); 2 Acque Industriali S.R.L., Via Bellatalla 1, Ospedaletto, 56121 Pisa, Italy; 3 Advanced Fibers, Empa Swiss Federal Laboratories for Materials Science and Technology, Lerchenfeldstrasse 5, 9014 St. Gallen, Switzerland; 4 Lab für Anorganische Chemie, ETH Zürich, HCI H 131 Vladimir-Prelog-Weg 1-5/10, 8093 Zürich, Switzerland

**Keywords:** deconvolution, spectral analysis, kinetic, xanthophyll, pigments, carotenoid, chlorophyll, EVOO, UV-visible absorption

## Abstract

Spectroscopic non-destructive methods have high potentialities as fast, cheap and easy-to-be-used approaches to address olive oil quality and authenticity. Based on previous research where near-UV Visible spectroscopy was used to investigate extra-virgin olive oils (EVOOs) and their main pigments’ content (i.e., β-carotene, lutein, pheophytin a and pheophytin b), we have implemented the spectral deconvolution method in order to follow the EVOO’s life, from ‘freshly pressed’ to ‘on-the-shelf’ EVOO samples at different storage time. In the first part of the manuscript, the new implemented deconvolution spectroscopic method aimed to quantify two additional pigments, namely chlorophyll a and chlorophyll b, is described and tested on ‘ad hoc’ samples with known concentrations of chlorophylls. The effect of light exposure and acidification was investigated to test the reliability and robustness of the spectral deconvolution. In the second part of the work, this approach was used to study the kinetic of pigments’ degradation in several monocultivar fresh EVOO samples under optimal storage’s conditions. The results here reported show that this spectroscopic deconvolution approach is a good method to study fresh EVOOs too; moreover, the proposed method revealed to be sensitive to detect eventual stresses of olive oil samples stored in not-good conditions.

## 1. Introduction

Virgin olive oil is an essential component of the Mediterranean diet and is, nowadays, an appreciated and recognized ingredient in many cultures. The increasing demand for extra virgin olive oils (EVOOs) is one of the reasons why these products are among the foods most susceptible to frauds. On the other hand, frauds in food and in particular in olive oils have a great impact on economic, ethic and social aspects, rising critical issues at different levels, from government to financial ones, of the modern societies [1]. There are several types of frauds involving the alteration of a food, such as the *sophistication*, consisting in adding substances that enhance the appearance or hide any food defect; the *adulteration*, consisting in replacing at least a component with one of lower value; the *alteration*, which is related to the degradation processes due to incorrect storage methods or a prolongation of storage times and the *counterfeit*, where brands or names of typical products are used in an inadequate way [2,3]. All the above frauds are usually intentional, and the main motivation is economical. For this reason, the research towards new methods to detect olive oil adulterations is important not only for its scientific impact, but also for economic and social impacts [4]. As regards extra virgin olive oil, numerous researches have been recently published in order to develop rapid and safe analytical methods to detect the presence of frauds and evaluate the genuineness of EVOOs on the market [5,6,7,8,9]. For instance, the adulterations of extra virgin olive oils with refined oils are usually discovered by measuring the amount of 1,2 diglycerides and 1,3 diglycerides, fatty acids’ relative percentages or other markers, while the sophistications made by adding walnut oils to EVOOs can be detected by the identification and quantification of several polar compounds, phospholipids, sterols and tocopherols [10,11,12]. In general, almost all chemical components can be considered useful to detect specific adulterations. Even pigments, which are present in EVOOs in a very low quantity (usually much less than 100 ppm), can be the subject of fraud. A well-known example is the addition of pigments, such as chlorophylls themselves (called E140) and copper complexes of chlorophyll derivatives (called E141). Several works are known concerning the detection of E 141i, which is liposoluble and also known as “copper chlorophylls”, and E 141ii, which is hydrosoluble and known as “copper chlorophyllins”, in olive oils [13,14,15,16]. Copper chlorophyllins (E 141ii) are the pigments most used in food processing, due to their hydrophilic character and because they generate a highly stable complex that remains green for a long-time during storage. The fact that E140 are allowed in some food matrices is at the basis of non-uniform regulations and diffuse use as adulterant in olive oil, too [1,15]. 

**Figure 1:**
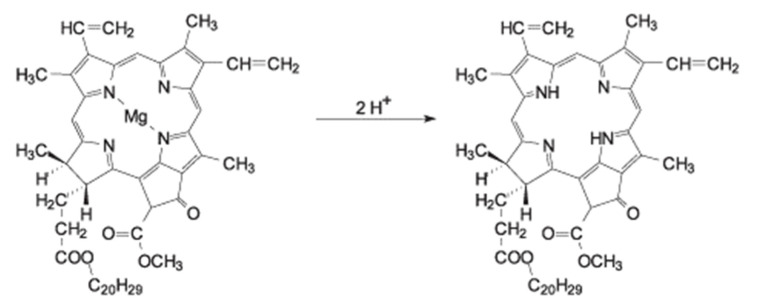
Process of transformation from chlorophyll a to pheophytin a, due to acidification.

The role of pigments is mostly related to the fact that pigments determine olive oil’s distinctive color. Their relative chemical composition varies during EVOO life, since it depends on many factors, such as the type of cultivar, the climatic/environmental conditions, the state of ripeness of the fruit at the time of harvesting, the olives’ sampling method, the oil production method, and the storage conditions of the EVOO final product [10]. Pigments can be divided in two main classes: carotenoids and chlorophylls with their derivatives [10,17,18,19,20,21]. Olive oils contain a relatively rich variety of carotenoids (i.e., β-carotene, lutein, violaxanthin, neoxanthin, and other xanthophylls in minor percentages) and chlorophylls with their derivatives (i.e., chlorophylls a and pheophytins a, chlorophylls b and pheophytins b, and other minor derivatives) [10,17,18,19,20,21]. Carotenoids are divided into two distinct classes of compounds: carotenes and xanthophylls. Some of them are precursors of vitamin A and their intake, through nutrition, promotes the reduction of diseases of the skin and eyes as well as the reduction of cardiovascular problems such as oxidation of low-density cholesterol (LDL) cholesterol [22]. For this reason, the interest in developing new methods to identify and quantify pigments is for human health, too. The structure of chlorophyll pigments is responsible for the absorption of the electromagnetic waves in the visible region of the spectrum of olive oils. Chlorophyll a has a blue-green color, while chlorophyll b is yellow-green. However, chlorophylls are very unstable compounds in the naturally acidic environment of the oil and therefore quickly undergo transformation processes. Only in “fresh oils” the chlorophyll content is still high [23,24]. Among the derivatives of the chlorophylls, the pheophytins are the most abundant: they derive from the substitution of the Mg^+2^ ion with two hydrogen ions (see Figure 1). 

The structural variation of the porphyrinic ring also causes the color of the oil to change, gradually passing from intense green to orange-yellow [25]. The transformation of chlorophylls into pheophytins, is a very important process that gives information both on the age of an oil and on its state of conservation. However, over time other degradation processes occur, such as the transformation of pheophytin a, in the absence of light and oxygen, in pyropheophytin a, an uncolored compound, for oxidative substitution of the carbomethoxy group by hydrogen [26]. Nowadays, the concentration of pyropheophytin a, is used as a quality parameter and it defines the aging state of an oil. 

In the literature, there are several researches about analytical methods able to identify and quantify pigments in oil matrices. Generally, the identification and quantification of single pigments is performed by means of chromatography, such as high-performance liquid chromatographic with ultraviolet-visible detection (HPLC-DAD) [18,19,20,21,26,27,28]. Near UV-vis spectroscopic absorption technique, on the contrary, has been used mainly to evaluate the total amount of carotenoids and chlorophylls’ derivatives from the absorbance values obtained, at single wavelengths, on olive oil samples diluted in a solvent, i.e., cyclohexane, as first reported by Mínguez-Mosquera et al. [18,29]. This simple and cheap spectroscopic method has been used in several works [18,29,30,31] to determine the total concentrations of carotenoids and chlorophylls in view of a chemical-physic characterization of olive oils. However, more recently, other methods based on the analysis of near UV-vis spectra of virgin and extra-virgin olive oils have been developed, either in combination with multivariate chemometric approaches or by using neural network to the spectral analysis [32,33,34,35,36,37,38,39]. These methods were optimized to check extra-virgin olive oil quality and EVOO authentication, however, a relatively complex data treatment and specific software are needed. On the other hand, the need for non-destructive and fast methods is justified by the raising competition in the field of extra-virgin and virgin olive oil and the consumers’ demand of products of high quality. 

For this reason, a recent new spectroscopic method, based on the quantitative analysis of the whole absorption spectrum of olive oil samples in the near UV-vis range, from 390 nm to 720 nm, has been developed. The method allows us to determine the concentration of four main pigments: β-carotene and lutein among the carotenoids, and pheophytin a and pheophytin b, among chlorophylls’ derivatives [38,39,40,41,42,43]. The advantage of this spectroscopic deconvolution method is the very fast analysis (about 1–2 min) and the absence of any sample treatment (any extraction or separative approaches). The absorption UV-vis spectra are indeed acquired in the bulk and then they are analyzed by using a spectral deconvolution (mathematical) procedure [38,44]. This spectroscopic approach has been already tested on extra-virgin olive oil samples produced in several Mediterranean countries (Spain, Greece, Italy and Tunisia), from different cultivars (such as *Leccino*, *Moraiolo*, *Pendolino*, *Frantoio*, *Chemlali*, *Poniente de Granada*, *Arbequina*, *Koroneijki*, *Cornicabra* and so on), and it was validated by comparing it with the other analytical methods [45,46], confirming its validity, goodness, and high reproducibility. Other studies mainly focused on monocultivar EVOOs produced in Tuscany (Italy) have shown the ability of this near UV-vis spectroscopic method to assess the high quality of EVOOs [47,48,49,50]. However, the main limitation of the method proposed by Domenici et al. [40] is related to the not satisfactory reproduction of near UV-vis spectra of fresh extra-virgin olive oils. In order to solve this limitation, in this work the method has been implemented by adding two pigments, namely chlorophyll a and chlorophyll b, present in freshly pressed olive oils [51,52].

In this paper, the implementation of the spectroscopic method with the addition of two more pigments for the spectral deconvolution, and several texts on ‘ad hoc’ samples as well as freshly pressed EVOO samples are reported. The effect of light exposure and acidification was investigated to test the reliability and robustness of the spectral deconvolution method. In the second part of the work, the implemented spectroscopic method has been used to study the kinetic of pigments’ degradation of several monocultivar EVOO samples under optimal storage and after light/thermal exposure. The results here reported show that the spectroscopic deconvolution approach is able to detect eventual stresses of olive oil samples stored in not-good conditions and to assess the quality of fresh extra virgin olive oils giving rise to a helpful tool to study and characterize EVOOs.

## 2. Materials and Methods

### 2.1. Reagents and Samples

Standard samples: chlorophyll a (CAS: 479-61-8) purity > 95% HPLC (impurity of chlorophyll b < 0.4%) and chlorophyll b (CAS: 519-62-0) purity > 90% HPLC (impurity of chlorophyll a < 0.5%) were purchased by Sigma-Aldrich (Milan, Italy). Solvents and reagents: acetone, triolein, methanol, n-hexane, hydrochloric acid and acetic acid were purchased by Sigma-Aldrich. 

EVOO samples used in this work are from Tuscany and from Apulia (Italy), two regions with an old tradition in the cultivation of *Olea Europaea* olive trees [53,54]. Tuscany adopts a still diffuse traditional cultivation, with typical cultivars *Leccino*, *Frantoio*, *Pendolino* and *Moraiolo*. Apulia region produces about 32% of all the olive groves in Italy, and it is the leading region for EVOO production in Italy [54]. EVOO samples selected for this study were produced, from different cultivars, in 2016 and in 2017. The label and basic information (cultivar, geographic origin, and day of oil production) of the EVOO samples analyzed in this work are reported in Table 1.

Fresh EVOOs (labels from A to G) were provided by local companies and they were obtained from different cultivars in 2017, as indicated in Table 1. Fresh EVOO samples were stored at T = 4 °C in the dark. Not fresh, here called ‘on-the-shelf’, EVOOs used in this work are four monocultivar EVOOs typical of Tuscany (Italy): *Frantoio*, *Leccino*, *Moraliolo*, and *Pendolino*. These samples (T1, T2, T3 and T4 labels) were produced by the local company S.S. Annunziata in Tuscany [55] from olive harvested in 2016 and they were stored far from light exposure, at T = 22 °C, in green bottles [52]. Refined olive oils used in this work were provided by Salov Spa (Massarosa, Italy) [40,43,47,51]. The EVOO samples selected for the study and reported in Table 1 can be considered quite representative of Italian EVOOs from the two regions, Tuscany and Apulia. 

### 2.2. Instruments

The UV-vis absorption spectra of the olive oil samples were acquired by using a UV-vis spectrophotometer (Jasco V-550, JASCO Europe Srl, Cremella, Italy) at room temperature. All experimental spectra of the olive oil samples in bulk were collected in the range between 220 and 800 nm with spectral resolution of 1 nm. All measurements on EVOO samples were performed with three replicates and the spectral analysis was performed in the region from 390 nm to 720 nm [40,41]. Depending on the samples, several types of quartz cells (Suprasil quartz) have been used with optical path of 1 cm, 0.5 cm, and 0.2 cm. Where it is not indicated, the optical path was 1 cm.

### 2.3. Implementation of the Deconvolution Method

The spectral deconvolution of UV-vis absorption spectra of olive oils is based on the assumption that the spectrum is due to the superposition of single absorption spectra of a discrete number of pigments present in EVOOs [40]. Within this assumption, the UV-vis spectrum, *ABS_calc_*(*λ*), can be calculated as a linear combination of the molar extinction spectra *ε_i_*(*λ*) of the *n* pigments (*i*, *j* = 1, 2, …, *n*).

*ABS*_*calc*_(*λ*) = *Σ_j_* × *C_j_* × *ε_j_*(*λ*) (1)

where the coefficient *C_j_* is the concentration of the *j*-pigment present in the sample. As reported in [40], it is easy to demonstrate that the coefficients *C_j_* can be obtained from a fitting procedure starting from the experimental UV-vis spectrum of the olive oil, *ABS_exp_*(*λ*), if the molar extinction spectra of the single pigments, *ε_i_*(*λ*), are known. Within the proposed mathematical approach, the superposition matrix *S*, calculated from the molar extinction spectra of single pigments, whose elements are defined as:

*S_i,j_* = ∫ *ε_i_*(*λ*) × *ε_j_*(*λ*) × *dλ* (2)

is first diagonalized and then, the eigenvectors *Φ_j_*(*λ*) and eigenvalues *α_j_* relative to each pigment are obtained. The concentration of the *r*-pigment, *C_r_*, is obtained as a linear combination of the coefficients, *γ_j_*, defined as:

*γ_j_* = ∫ *Φ_i_*(*λ*) × *ABS_exp_*(*λ*) × *dλ*/*α_j_* (3)

The first version of the deconvolution method [40,41,42,43,44,45,46,47] was designed, tested, and validated on a large set of ‘on-the-shelf’ EVOOs produced from different cultivars, in different geographic areas and harvesting years. The first deconvolution model includes four pigments (lutein, β-carotene, pheophytin a, and pheophytin b), which can be safely assumed to represent more than 95% of the pigments’ content of ‘on-the-shelf’ EVOOs and, in general, of ‘not-fresh’ virgin and extra virgin olive oils [10,16,17,18,19,40,41,42,43,44]. Moreover, this model includes the spectrum of triolein, which represents a sort of base-line, chosen after several tests [40,41,42] to reproduce the olive oil matrix. The molar extinction spectra of the four pigments, *ε_i_*(*λ*), were indeed obtained experimentally by diluting known amounts of each pigment in triolein [40,41].

**Figure 2:**
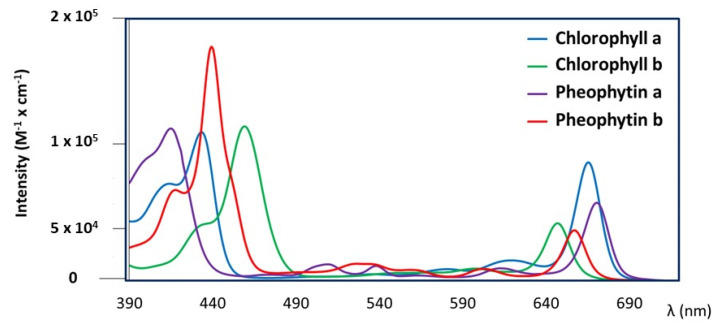
Molar extinction coefficients, *ε_i_*(*λ*), of four pigments diluted in triolein: chlorophyll a, chlorophyll b, pheophytin a, and pheophytin b.

In an analogous way, in the present work, the molar extinction spectra of chlorophyll a and chlorophyll b were obtained. The standard of chlorophyll a was diluted in acetone, while the standard of chlorophyll b was diluted in methanol. The concentration of the two solutions, *C_i_* (*i = a* for chlorophyll a and *i = b* for chlorophyll b), was determined from the UV-vis absorption spectrum, by using the molar extinction coefficients present in the literature relative to the appropriate solvents [56,57], by using the Lambert–Beer law:

(4) $$C_{i}=\frac{ABS_{i}\left(\lambda \right)}{\varepsilon_{i}\left(\lambda \right)\times l}$$

with *l* = 1 cm. The obtained concentrations are *C_a_* = 6.33 × 10^−5^ M and *C_b_* = 5.63 × 10^−5^ M, respectively. A precise volume (0.700 mL) of each solution was dried; the residue was than diluted in 0.700 mL of pure triolein. The molar extinction coefficients of the two pigments in triolein, *ε_a_*(*λ*) and *ε_b_*(*λ*), were determined from the experimental UV-vis absorption spectra of the two solutions of chlorophyll a and chlorophyll b in triolein and they are reported in Figure 2 together with the molar extinction coefficients of pheophytin a and pheophytin b in triolein, previously obtained [40].

The new two molar extinction coefficients of chlorophyll a and chlorophyll b were included into the new deconvolution model which was used, first, to analyze the spectra of ‘ad hoc’ samples, with known concentrations of pigments, and, second, to study real olive oil samples, fresh EVOOs and to investigate the kinetics of pigment degradation in monocultivar EVOO samples. 

### 2.4. Data Analysis

Spectral data were analyzed by using home-made sheets of Microsoft Office Excel 2016 (Microsoft Corporation, Redmond, Washington, DC, USA). The goodness of the mathematical deconvolution method was verified by the R-square (*R*^2^) test, which estimates the correlation between the experimental spectrum and the calculated one by the deconvolution procedure:

(5) $$R^{2}=1-\frac{\sum_{i=1}^{n}{\left(y_{i}-f_{i}\right)}^{2}}{\sum_{i=1}^{n}{\left(y_{i}-\bar{y}\right)}^{2}}$$

where *f_i_* is the value predicted by the fitting, $\bar{y}$ is the mean of the observed data and *y_i_* is the observed data value.

The concentrations obtained in this work from the spectral deconvolution method are expressed as mean value ± standard deviation. The spectral analysis was indeed performed in triplicate: for each sample three spectra were acquired and then analyzed through the spectral deconvolution model giving rise to best fitting results in terms of concentration of pigments. 

Kinetic evolution of pigments in EVOOs was studied by using a home-made program working on Mathematica 5.2 (Wolfram, Champaign, IL, USA). 

## 3. Results and Discussion

### 3.1. Application of the Spectroscopic Method on Test Samples 

#### 3.1.1. Chlorophylls Diluted in Refined Oil

**Figure 3:**
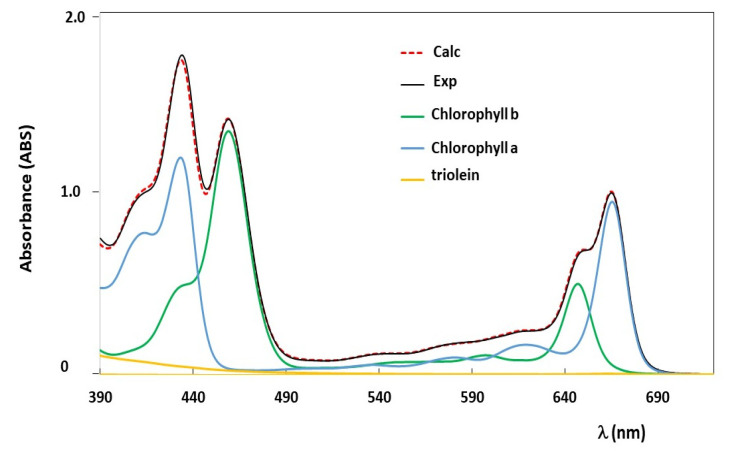
Experimental (Exp) and theoretical (Calc) UV-vis absorption spectra of a test sample “CAB_15_15” prepared with chlorophyll a (15 ppm) and chlorophyll b (15 ppm) diluted in a refined oil. Wavelengths are reported in nm. The optical length was of 0.5 cm. Single pigments’ spectral contributions obtained from the deconvolution analysis are also reported, namely chlorophyll a and chlorophyll b. The spectral base-line due to the oil matrix is reported as ‘triolein’ spectral contribution.

Several tests of the new implemented deconvolution method were performed on samples prepared ‘ad hoc’. Known amounts of chlorophylls were diluted in precise volumes of a refined oil produced by Salov Spa (see Section 2.1). Before proceeding with the preparation of these samples, the UV-vis spectrum of the refined oil was registered to prove the absence of any pigments or other compounds absorbing in the region from 390 nm to 720 nm, which is the spectral window of interest. Samples were prepared with single pigments, namely chlorophyll a (sample label CA_n) or chlorophyll b (sample label CB_m, or with both (CAB_n_m), where the two index ‘n’ and ‘m’ stay for the values of concentration, expressed in ppm, of chlorophyll a and chlorophyll b, respectively. UV-vis spectra of all prepared samples were recorded as reported in Section 2.2 and they were analyzed by using the deconvolution model. An example of UV-vis absorption spectrum of the CAB_15_15 sample is reported in Figure 3. The calculated UV-vis spectrum obtained from the deconvolution analysis as well as the triolein baseline and single pigments’ contributions, namely chlorophyll a and chlorophyll b, are displayed too.

The values of the concentrations, *C_i_*, obtained from the deconvolution of the experimental UV-vis spectra of all prepared test samples are reported in Table 2, together with the values of *R*^2^. 

Data reported in Table 2 show a very good agreement between the obtained values of concentrations and the known concentrations (see samples’ labels). Moreover, as also shown in Figure 3 for the CAB_15_5 sample and from the value of *R*^2^ reported in Table 2, the calculated UV-vis spectra well reproduce the experimental ones. 

#### 3.1.2. Degradation of Chlorophylls into Pheophytins

A further test of the new implemented method was performed by following several degradation processes of the pristine ‘ad hoc’ samples prepared as described in the previous section. Several test samples (CA_n and CB_m) were indeed prepared by diluting known amounts of chlorophyll a or chlorophyll b in precise volumes of a refined oil. These samples were stored in transparent or in green glass bottles and left at room temperature for several hours or days, depending on the type of degradation experiment and kinetic rate. Some of these representative experiments and relative results are reported in the Supplementary Materials (Figures S1–S3). In all experiments, the analysis of the UV-vis spectra at different times revealed the progressive degradation of both chlorophylls due to the effect of light and the consequent decreasing of the concentration of chlorophylls (see for instance Figures S1 and S2 in the Supplementary Materials). Here, the spectral reproduction of the experimental UV-vis spectra was very good (*R*^2^ > 0.994). 

An additional experiment was performed in order to check the goodness of the method in reproducing the UV-vis experimental spectra in case both chlorophylls and pheophytins were present. Two test samples (CA_20.5 and CB_21.1) were prepared and inserted into a green glass bottle (sample volume of 1.4 mL). In order to accelerate the process of pheophytinization, namely the transformation of chlorophylls into pheophytins, as described in Section 1, several microliters of acetic acid were added in two steps, reaching the pH of 4. 

**Figure 4:**
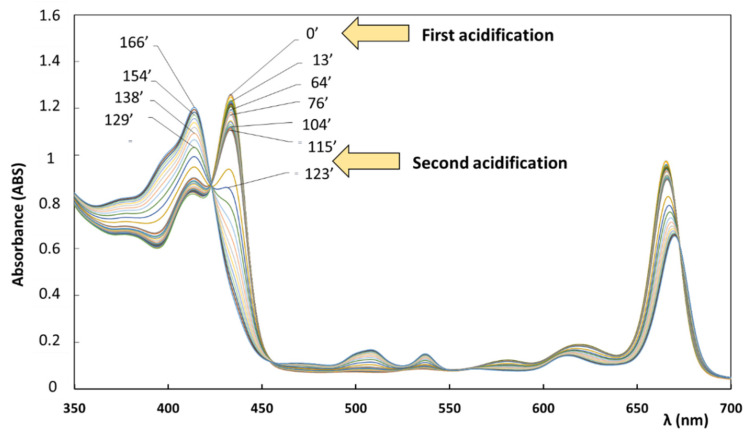
Evolution of the UV-vis absorption spectrum of a test sample (‘CA_20.5’) containing chlorophyll a diluted in refined oil, after a first (at time = 0′) and a second (at time = 117′) acidification, as described in the text. Spectra were recorded during time, from 0 to 166 min, following the complete conversion from chlorophyll a to pheophytin a. The optical path was 0.5 cm.

Under these conditions, as reported in the literature [58], chlorophylls evolve into pheophytins much faster than in ‘normal’ conditions. Moreover, in order to limit the eventual formation of pyropheophytins and other uncolored derivatives, the samples were stored in the dark and the temperature was monitored during the experiment not to be higher than 18 °C. In Figure 4, several UV-vis absorption spectra recorded on the CA_20.5 sample at different times, expressed in minutes (′), are reported. As shown in the figure, two acidifications were performed by adding acetic acid at time 0′ (4 mL) and at time 120′ (6 mL). The UV-vis spectra recorded during time and reported in Figure 4 clearly show the evolution from a spectrum characterized by the presence of chlorophyll a, as unique pigment, to a spectrum due to the presence of both chlorophyll a and pheophytin a. The complete conversion of chlorophyll a into pheophytin a is observed after 166′ (see Figure 4). All recorded spectra were then analyzed by using the new deconvolution model and the concentration of both chlorophyll a and pheophytin a was determined from the fitting analysis of each spectrum. 

**Figure 5:**
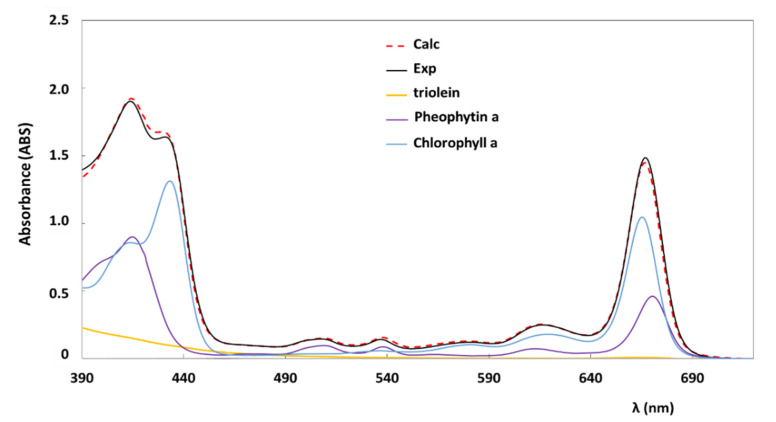
Experimental (Exp) and theoretical (Calc) UV-vis absorption spectra of the test sample (‘CA_20.5’) at time = 123′ during the pheophytinization process (see Figure 4). Single pigments’ spectra used in the deconvolution are also reported, namely chlorophyll a and pheophytin a. Triolein spectral contribution is also shown. Optical path was 0.5 cm. The value of *R*^2^ of the deconvolution was 0.997.

As an example, in Figure 5, the experimental spectrum recorded at time 123′ and obtained from the fitting procedure reported a very good reproduction of the spectrum even in the case of simultaneous presence of comparable amounts of pheophytin a (value determined from the fitting, C = 7.75 ppm) and chlorophyll a (value determined from the fitting, C = 11.6 ppm). 

The values of concentration of chlorophyll a and pheophytin a obtained at different times from the fitting of the experimental spectra are reported in the Supplementary Materials (Table S1). The goodness of the fitting was evaluated by the R-square test, showing values of *R*^2^ higher than 0.996 for all the spectra reported in Figure 4. Analogous experiments were performed in the case of the sample CB_21.1, prepared with a known concentration of chlorophyll b diluted in a refined oil (see Table S2 in the Supplementary Materials). In the two experiments the process of pheophytinization were accelerated by adding small amounts of acetic acid. These tests were useful to verify the ability of the new implemented method to analyze samples with contemporaneous presence of chlorophylls and pheophytins. The results were very satisfactory in both samples.

### 3.2. Application of the New Method on Real Extra-Virgin Olive Oil Samples

#### 3.2.1. Spectral Analysis of Fresh and ‘On-the-Shelf’ EVOOs

**Figure 6:**
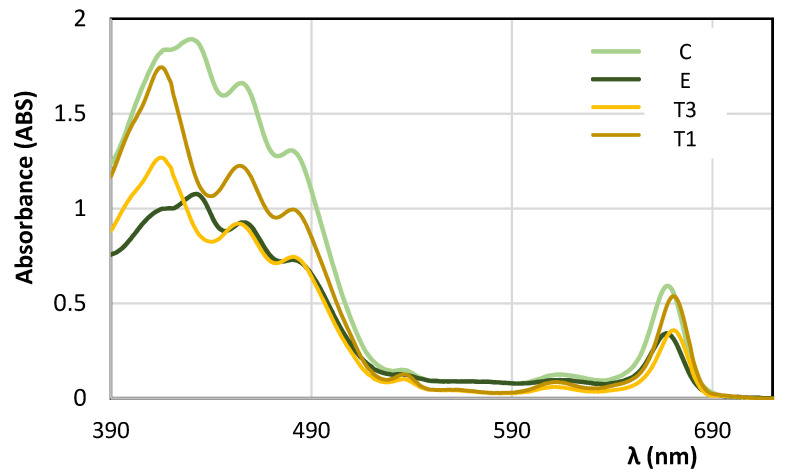
Experimental near UV-vis spectra of fresh EVOOs (samples C and E) and ‘on-the-shelf’ EVOOs (sample T1 and T3). Spectra were recorded after 30 and 38 days from the oil production in the case of sample C and E, respectively. On-the-shelf EVOOs were analyzed after ~4 months from their production. Spectra were recorded with optical path of 0.5 cm.

Once the new method for spectral deconvolution has been tested on samples produced ad hoc with known amounts of chlorophylls, several EVOO samples have been selected to perform further studies on real samples. As demonstrated in other works [40,41,42,43,44,45,46,47,48], EVOOs present on the market after several months from their production contain neglectable amounts of chlorophylls, since most of them are converted in pheophytins and other not colored chlorophylls’ derivatives [26,59]. If we compare UV-vis absorption spectra of fresh EVOOs with those of ‘on-the-shelf’ EVOOs, several differences can be observed at a qualitative level, too. In Figure 6, UV-vis absorption spectra of two fresh EVOOs (samples C and E) after about 1 month from their production, stored in the dark at 4 °C, are shown. These samples have been selected since their pigments’ content was rather different: sample C contains about 30 ppm of total pigments, while sample E has quite a low pigment content (about 16 ppm). Despite of this difference, the shape of the two spectra is similar and it is characterized by a band between 390 and 500 nm with a four-peaks shape and higher absorbance at 430 ppm, and a peak at 666 ppm, typical of chlorophyll a. In the same figure, the near UV-vis absorption spectra of two “on-the-shelf” EVOOs (samples T1 and T3) stored at 22 °C, registered after about 4 months from their production, are shown. As it can be observed from Figure 6, the two spectra of not-fresh EVOOs are characterized by a band between 390 and 500 ppm with the typical ‘three-peaks’ shape and higher absorption at 416 ppm, and a peak centered at 671 ppm, typical of pheophytin a [40,60].

**Figure 7:**
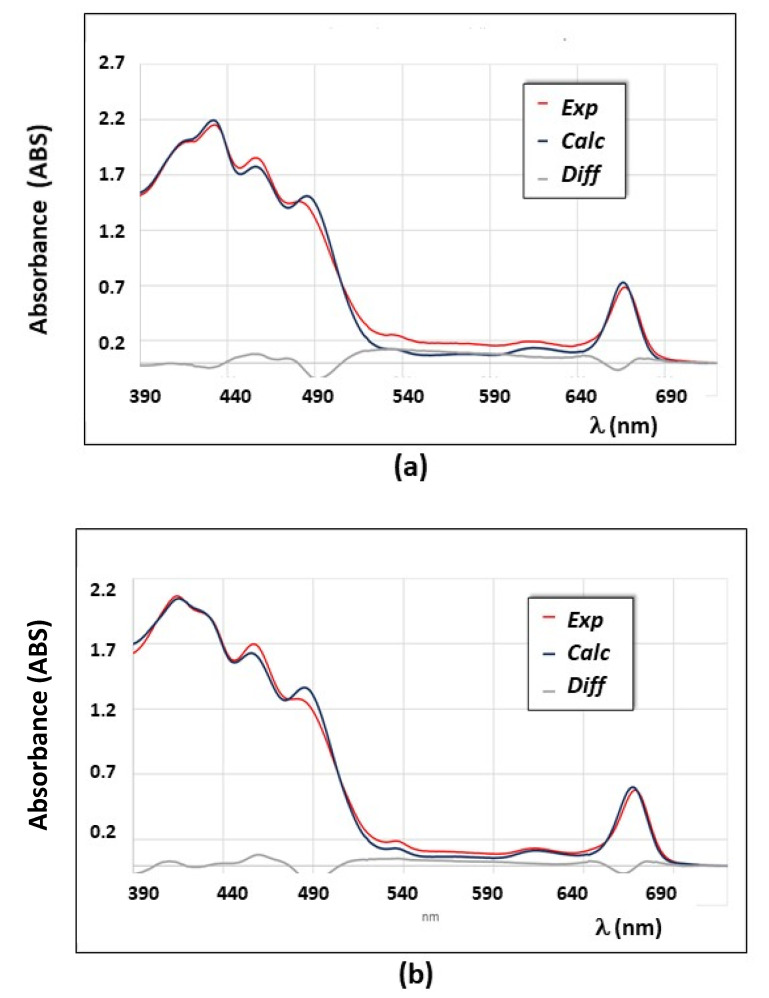

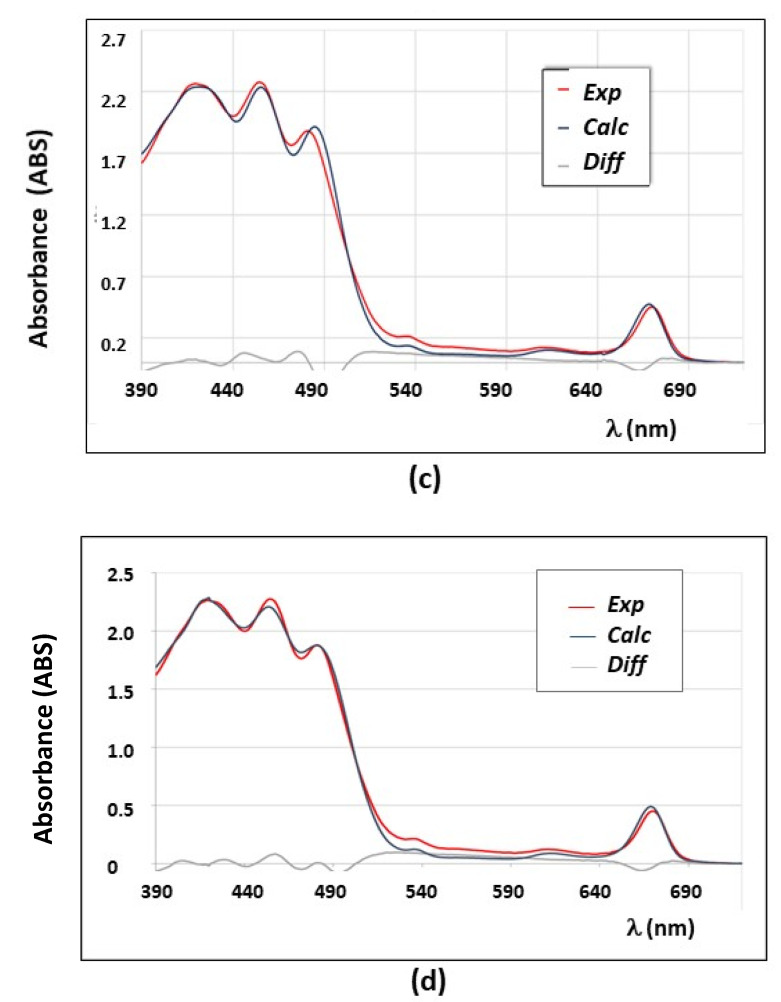
Experimental (Exp) and calculated (Calc) UV-vis absorption spectra of fresh EVOO samples. Residues are also show in the plot (Diff). Spectra are here reported with an optical path of 1.0 cm. (**a**) Sample E (after 35 days from oil production); (**b**) sample E (after 95 days from oil production); (**c**) sample F (after 40 days from oil production); (**d**) sample B (after 38 days from oil production). Data analysis was performed through the new deconvolution model, as described in the text.

The spectral differences among fresh and ‘on-the-shelf’ EVOOs are due to the different types of pigments and their relative concentration. In particular, as demonstrated in detail in previous works [40,41,42,43,44,45,46,47,48], UV-vis spectra of not-fresh EVOOs can be reproduced almost exactly with the deconvolution model [40] by using four main pigments: β-carotene and lutein, among carotenoids, pheophytin a and pheophytin b, among chlorophylls’ derivatives. In the case of fresh EVOOs, as seen in Figure 6, chlorophylls are obviously present and the spectral analysis needs to be performed by including the deconvolution model into the chlorophylls’ molar extinction spectra, shown in Figure 2. Several examples of the best deconvolution spectral analysis obtained with the new model, implemented as described in Section 2.3, on fresh EVOOs are reported in Figure 7.

The spectral deconvolution analysis of sample E, recorded at different times after the olive oil production is reported in Figure 7a,b. As it can be seen, the spectral shape changes significantly between 390 and 440 nm due to the progressive conversion of chlorophylls into pheophytins. The best fitting analysis is quite satisfactory even though it is not perfect in the spectral area of the two relative peaks around 458–460 nm and 485–488 nm. Other examples of spectral deconvolution results are reported for sample F (Figure 7c) and sample B (Figure 7d), recorded after ~40 days from their production: in this last case, the spectral reproduction is almost perfect as it can be seen from the residues, displayed in the figure, too. As also observed for the all fresh EVOO samples analyzed in this work, within 30–40 days from their production, the spectra are well reproduced by using the following as main pigments: β-carotene and lutein, among carotenoids, and chlorophyll a and pheophytin a, among chlorophylls’ derivatives. The amount of chlorophyll b and pheophytin b is in all cases very small (less than 0.5 ppm) and their use in the deconvolution model does not affect the spectral analysis significantly. Moreover, quantities of pigments below 0.5 ppm are comparable with the total error associated to the total amount of pigments by using this method, as shown in Table 3. For this reason, the analysis of pigments’ content and the study of kinetics of pigments in fresh EVOOs by using the spectroscopic method, is performed without including neither chlorophyll b or pheophytin b.

In Table 3, the results obtained from the spectral analysis for all analyzed fresh EVOOs (see Table 1) after about 1 months from their production is reported showing the concentration of the four main pigments and the total amount of pigments, expressed in ppm. The standard deviation for each value of concentration is also reported, calculated as described in Section 2.4. The value of *R*^2^ is very close to 1 (>0.995).

As it can be seen in Table 3, the fresh EVOO samples under investigation have a different content of total pigments, ranging from about 30 ppm (sample C) to about 17 ppm (sample E). The relative pigments’ concentration, for instance the ratio between carotenoids and chlorophylls’ derivatives, is also different from sample to sample due to the different EVOO characteristics (i.e., the geographic area of olives’ production and olives cultivars). Moreover, even though these samples were stored in the dark at T = 4 °C, and the spectra were acquired after about 40 days from the oil production, the conversion from chlorophyll a to pheophytin a is already started. Interestingly, in some cases (samples A, B and D) the ratio between chlorophyll a and pheophytin a is close to 1 (less than 1.5), while in the other cases, this ratio is higher, as in the case of sample E (ratio ~2.1), thus suggesting that small differences in the kinetic processes among these samples is expected. In the next section, the evolution of pigments in several ‘on-the-shelf’ EVOOs and in fresh EVOOs is analyzed in terms of kinetic processes. The pigments’ concentration at different times was determined in all cases by using the spectral deconvolution model.

#### 3.2.2. Kinetic Study of Pigments in ‘On-the-Shelf’ and Fresh EVOOs

In this section we are reporting the study of evolution of pigments’ concentration in two sets of samples: ‘on-the-shelf’ monocultivar EVOOs (samples T1, T2, T3, and T4) and fresh EVOOs produced in different areas (Tuscany and Apulia, Italy) from different cultivars (samples from A to G).

Among ‘on-the-shelf’ EVOOs the set selected for the kinetic study was produced by the same farmer [55] from four different cultivars in Tuscany (Italy). The geographic and climate conditions were exactly the same, and the day of oil production was very close (see Table 1). These samples were stored under light and temperature conditions similar to those of the shelves in the markets. In particular, the four EVOO samples were stored in green glass bottles at 22 °C on the shelf in the laboratory. One of the advantages of using the spectroscopic method to determine the pigments’ concentrations is that the analysis can be performed exactly on the same sample, as was achieved in the present study. The typical spectrum of not-fresh EVOOs is reported as an example in Figure 6: see the spectra of samples T1 and T3 registered after about 4 months from their production.

The main pigments’ concentrations of the four samples (T1, T2, T3 and T4) determined by using the spectral deconvolution model [40,41,42,43,44,45,46,47,48] with four pigments (β-carotene, lutein, pheophytin a, and pheophytin b) are reported in Table 4.

The fittings of the experimental spectra of not-fresh EVOOs are very good, as indicated by the values of *R*^2^, and in agreement with previous studies where the deconvolution method with four pigments (β-carotene, lutein, pheophytin a and pheophytin b) was introduced and tested on several EVOOs [40,41,42,43,44,45,46,47,48]. In the present work, the same samples (T1, T2, T3 and T4) were analyzed in a long period (from about 80 days to 800 days after their production) without changing their storing conditions.

**Figure 8:**
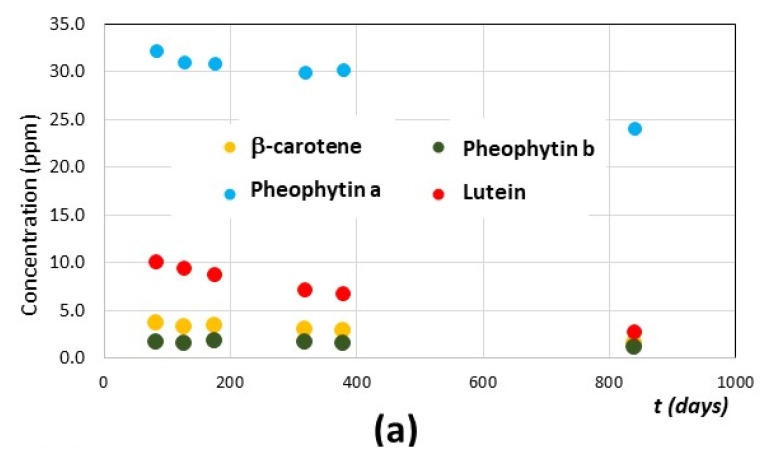

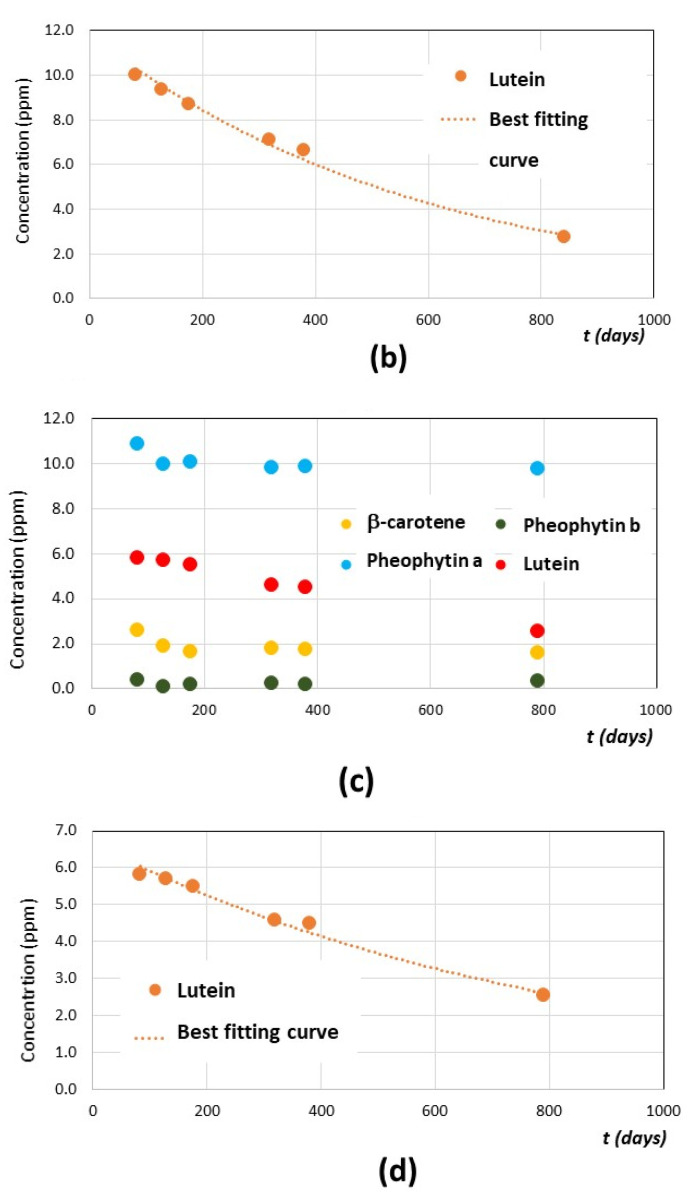
(**a**,**c**) Trends of the concentrations of four main pigments (β-carotene, lutein, pheophytin a and pheophytin b) in two EVOO samples, stored in the dark at T = 22 °C, during time (from about 80 to 800 days after their production): (**a**) a monocultivar *Moraiolo* EVOO (sample T2) and (**c**) a monocultivar *Pendolino* EVOO (sample T4). (**b**,**d**) Values of the concentration (ppm) of lutein during time (t), expressed in days. Symbols refer to values obtained from the deconvolution of the experimental spectra and dashed curves represent the best fitting from exponential analyses (see Equation (7)), as described in the text. (**b**,**d**) refer to sample T2 and sample T4, respectively.

As known in the literature [17,24,58,59,60], if EVOOs are not affected by light exposure, temperature shock, or oxygen treatment; the degradation of pigments is quite slow and the whole quality of the EVOOs can be preserved for more than 10 months after their production. The trends of concentrations (ppm) versus time (expressed in days) of the four pigments in T2 and T4 EVOO samples are shown in Figure 8a,c, while the data for all four samples (T1, T2, T3 and T4) are reported in the Supplementary Materials (Table S3). 

Error bars are smaller than symbol size. As noted in Figure 8 and Table S3, the amount of pheophytin b is very low and its variation during time is not significant. Pheophytin a, which represents the most abundant pigment in these four EVOO samples, shows a slow decrease, which becomes significant only for the sample T2 (*Moraiolo* cultivar). In the other samples, pheophytin a remains almost constant even after about 2 years from oil production. 

In the case of carotenoids, β-carotene content decreases in all EVOOs until ~120 days from oil production and then it remains almost constant during the period of investigation (about 2 years). Lutein, the most abundant carotenoid, shows a progressive decrease during time for the four EVOOs.

Kinetics of degradation of lutein is here reported as an example: in Figure 8b,d the trends of concentration of lutein in samples T2 and T4, respectively, are shown superimposed with the best fitting curves obtained by applying a first order kinetic [17,60]. Within this kinetic model, the concentration of the i-pigment, $\left[C_{i}\right]$, varies during time according to Equation (6), where $k_{i}$ is the kinetic constant of degradation:

(6) $$\frac{d\left[C_{i}\right]}{dt}=-k_{i\;}\times \left[C_{i}\right]$$

In order to verify the first order process of degradation, it is useful to evaluate the instant rate of degradation according to Equation (7), and to plot the value of $P_{oi}$, expressed as in Equation (8), as a function of the initial concentration $\left[C_{i}\right]$_0_.

(7) $$\frac{d{\left[C_{i}\right]}_{t}}{dt}=-k_{i}\times {\left[C_{i}\right]}_{0}\times e^{-k_{i}t}$$

(8) $$P_{oi}=-k_{i}\times {\left[C_{i}\right]}_{0}$$

In Table 5, the values of initial concentration of lutein for the four samples, [*C*]_0_, the kinetic constant, *k*, and value of *P*_0_, are reported, as obtained from the fitting of the experimental trends of concentrations reported in Figure 8 and Table S3. In all cases, the goodness of the fitting was evaluated according to the R-square method, reaching values between 0.9881 and 0.9958.

As seen in Table 5, three samples have the same value of the kinetic constant (T1, T3, and T4), while sample T2 has a constant rate double than the other samples. A possible explanation is related to the much higher pigments’ content of sample T2 with respect to the others, which may affect the degradation mechanisms and their rates. In all cases, the obtained values of kinetic constant at 22 °C are coherent with those reported in the literature for several carotenoids present in EVOO and VOO samples under similar thermal conditions [59,60].

**Figure 9:**
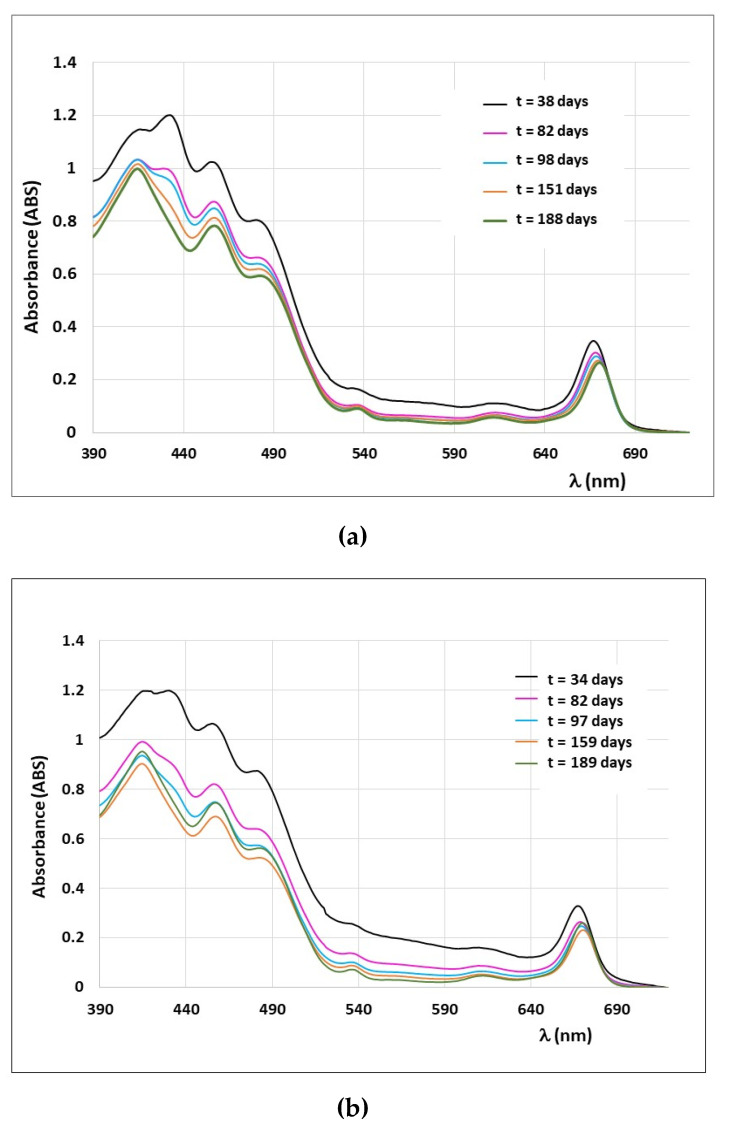
Experimental UV-vis absorption spectra of fresh EVOO sample F (**a**) and sample B (**b**) recorded with a quartz cuvette (optical path of 0.5 cm) at different times. Spectra of different color were recorded at different times, as indicated in the legend: ‘t’ stays for the number of days after the olive oil production.

In order to further test the deconvolution model applied to fresh EVOOs, a kinetic study was performed on several fresh EVOO samples produced in Italy (see samples A-G in Table 1). These samples were stored in the dark at T = 4 °C and the same sample volumes were analyzed in a period of about 4 months. In Figure 9, the evolution of the spectra during time is reported as an example for sample F (Figure 9a) and sample B (Figure 9b).

As it can be seen in Figure 9, the spectral shape of fresh EVOOs changes significantly from the first spectrum (recorded after 30–40 days from olive oil production) to the second spectrum (recorded after 85–90 days from olive oil production). These changes involve both the absorbance intensity and the spectral shape. At times longer than 85–90 days, the spectra change mainly in the spectral shape, revealing a variation of the relative pigments’ content. In all cases, the experimental spectra are analyzed by using the deconvolution model with four pigments: β-carotene and lutein, among the carotenoids, and chlorophyll a and pheophytin a, among the chlorophylls’ derivatives. The spectral reproduction is satisfactory for all spectra recorded at different times, with a value of *R*^2^ always higher than 0.990. 

**Figure 10:**
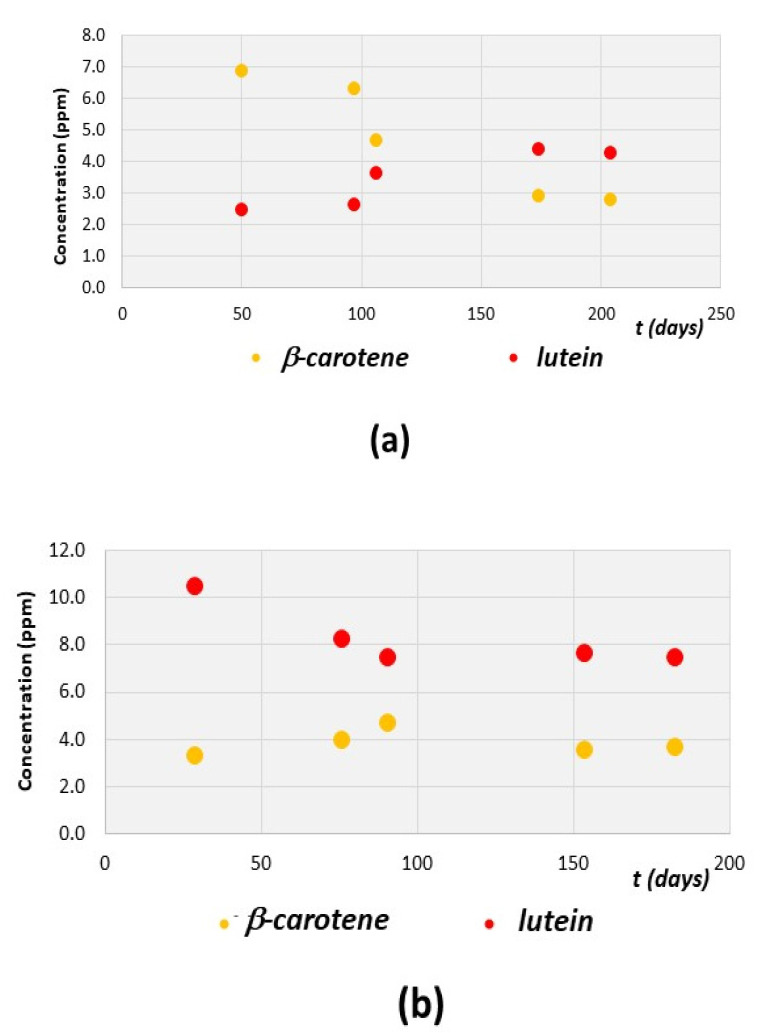

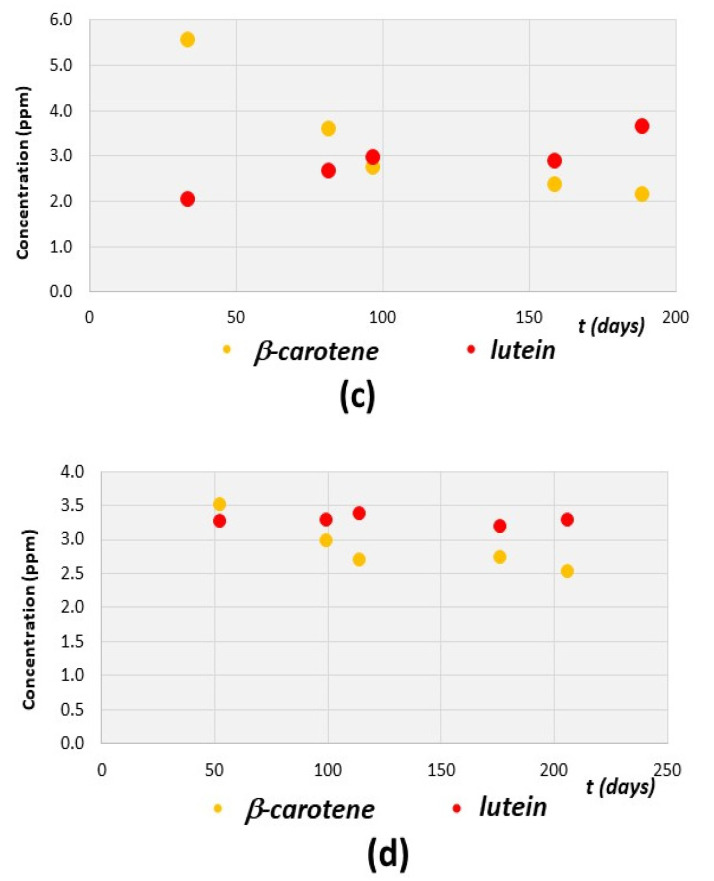
Trends of the concentration (ppm) of β-carotene (yellow circles) and lutein (red circles) during time (t) expressed in days of several fresh EVOO samples. Data are obtained from the deconvolution of the experimental spectra. Data refer to (**a**) sample A, (**b**) sample C, (**c**) sample D, and (**d**) sample E.

As an example of evolution of the pigments’ content, the cases of fresh EVOO samples A, C, D, and E are here reported in details. Samples A and D were selected since they have a relatively high initial pigments’ content (see Table 3) and they were produced in Tuscany and Apulia, respectively, from selected cultivars (see Table 1). Samples D and E are both blend EVOOs produced in Tuscany in the same location and from the same olive trees (with prevalence of *Moraiolo* cultivars). These samples have a relatively low initial pigments’ content with a slightly different pigments’ relative concentration. The spectral deconvolution of the analyzed fresh EVOO samples reveals a different behavior of the two main carotenoids, namely β-carotene and lutein, depending on the sample (see Figure 10).

**Figure 11:**
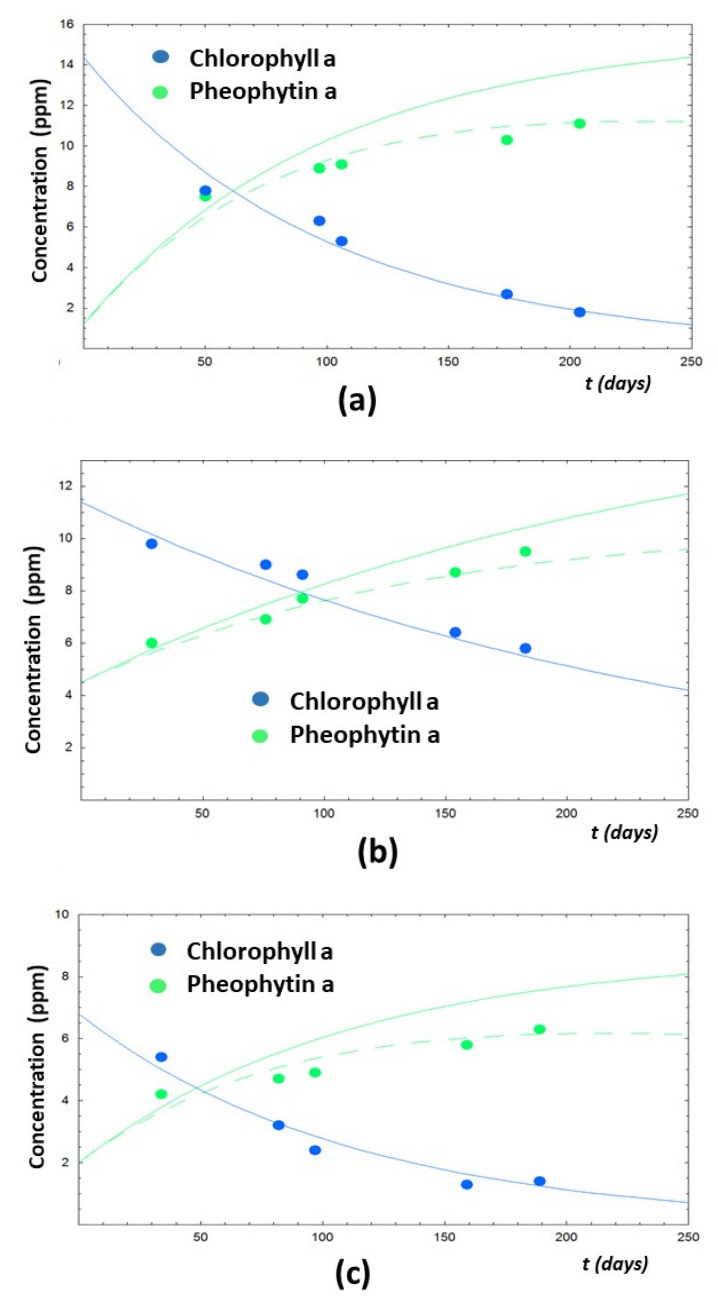

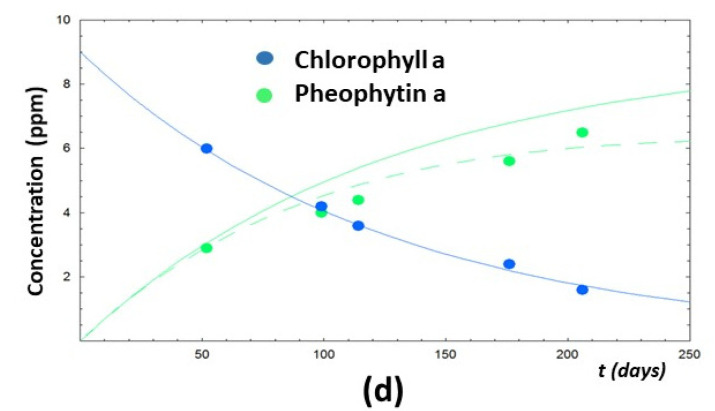
Trends of the concentration (ppm) of chlorophyll a (blue circles) and pheophytin a (green circles) during time (t) expressed in days of several fresh EVOO samples. Data are obtained from the deconvolution of the experimental spectra. Data refer to (**a**) sample A, (**b**) sample C, (**c**) sample D, and (**d**) sample E. Solid curves and dashed curves are best fitting according to the two models expressed with Equations (9)–(12), respectively.

Two EVOO samples (A and D) have an initial amount of β-carotene much higher than lutein (at ~40 days from oil production). Moreover, in both samples, β-carotene concentration decreases during time, while lutein concentration increases during time. An almost opposite trend is found in sample C, even though the concentrations of β-carotene and lutein remain almost constant at t > 100 days. In the case of sample E, the carotenoids’ content does not increase during time so much, as noted in Figure 10d. The explanation of the different behaviors of carotenoids’ concentration in the selected EVOO samples is still not clear, but it could be related to the not-perfect fitting of some spectra in the region between 440 and 540 nm, which is ascribable to the carotenoids’ content (see for instance Figure 7). On the contrary, the trends of chlorophyll a and pheophytin a during time are similar for all fresh EVOO samples analyzed in this work. In particular, as expected, chlorophyll a concentration decreases during time and that of pheophytin a increases (see Figure 11), with trends that can be analyzed in terms of degradation processes and kinetic models.

As reported in the literature, the degradation mechanisms affecting chlorophylls and their derivatives are quite complex, however the kinetics of these processes can be modeled as first order kinetics [26,58]. The simplest model consists in considering the degradation of chlorophyll a into pheophytin a, without further degradation processes. In such a case, a single kinetic constant, k_1_, can be used to fit the experimental data. The time evolution of the concentration of chlorophyll a, [C_a], and pheophytin a, [Ph_a], according to this simple model are reported in Equations (9) and (10):

(9) $${\left[C\_a\right]}_{t}={\left[C\_a\right]}_{0}\times e^{-k_{1}t}$$

(10) $${\left[\mathrm{Ph}\_a\right]}_{t}={\left[\mathrm{Ph}\_a\right]}_{0}+{\left[C\_a\right]}_{0}\times \left(1-e^{-k_{1}t}\right)$$

where [C_a]_0_ and [Ph_a]_0_ represent the concentrations of chlorophyll a and pheophytin a at time t = 0, respectively.

A slightly more complex model implies the presence of two main degradation processes: (1) the transformation from chlorophyll a to pheophytin a (with kinetic constant k_1_) and (2) the further transformation of pheophytin a to not-colored derivatives, such as pyropheophytin a [26], (with a global kinetic constant k_2_). The evolution of the concentrations of these two pigments with time can be expressed as:

(11) $${\left[C\_a\right]}_{t}={\left[C\_a\right]}_{0}\times e^{-k_{1}t}$$

(12) $${\left[\mathrm{Ph}\_a\right]}_{t}=\frac{{\left[C\_a\right]}_{0}\times {\;k}_{1}}{\left(k_{2}-k_{1}\right)}\times \left({\;e}^{-k_{1}t}-e^{-k_{2}t}\right)+\;{\left[\mathrm{Ph}\_a\right]}_{0}\times e^{-k_{2}t}$$

The two models of kinetic degradation have been used to analyze the trends of chlorophyll a and pheophytin a in the four selected fresh EVOOs during time as reported in Figure 11. The second model allowed us a better reproduction of the time evolution of pheophytin a with respect to the simplest model (see green solid versus green dashed curves in Figure 11).

The best fitting values of initial pigments’ content and kinetic constants, k_1_ and k_2_, are reported in Table 6 for the four EVOO samples. 

The fitting of the experimental trends of chlorophyll a and pheophytin a are satisfactory and the values of kinetic constants are in the same range of those reported in ref. [26], where the concentrations of pigments in several Spanish EVOOs were determined by means of standard HPLC methods. As reported previously [26], the degradation rate of chlorophyll a is faster than that of pheophytin a in all cases analyzed, except that in sample D, where the two kinetic constants are similar.

The differences among the four fresh EVOO samples may be explained in terms of initial concentrations (at time t = 0) of the two pigments, which may be related to the olive cultivars and maturation index at harvesting time [52,53,54,55,56,57,58]. The fact the kinetic model developed for chlorophylls’ derivatives degradation under different storage conditions [26] well fits with our data is a further confirmation of the goodness of the spectroscopic deconvolution method to study fresh EVOO samples.

## 4. Conclusions

In this work, the spectral deconvolution method, previously proposed to quantify main pigments, namely β-carotene, lutein, pheophytin a, and pheophytin b, in EVOOs from their near UV-visible spectra in the bulk [40,41,42,43,44,45,46,47,48], was implemented with two additional pigments: chlorophyll a and chlorophyll b. Several examples of application of the new implemented spectroscopic method are here presented. First, several tests were performed on samples prepared with known amounts of chlorophyll a and chlorophyll b diluted in refined oil. These samples were also analyzed during storage, after light exposure and acidification, at different times. The reproduction of the spectra was very satisfactory. The new spectroscopic method allowed us to follow the time evolution of the chlorophylls’ degradation as well as the process of pheophytinization. In the second part of the paper, near UV-vis absorption spectra of EVOO samples produced in Italy (Tuscany and Apulia), both fresh and ‘on-the-shelf’ ones, were analyzed in terms of pigments content. These EVOO samples can be considered quite representative of Italian EVOOs, since they were produced by different cultivars typical of these two regions. Confirming previous results [40,41,42,43,44,45,46,47,48], spectra of ‘on-the-shelf’ EVOOs can be well reproduced with four pigments: β-carotene, lutein, pheophytin a and pheophytin b. A limitation of the original method is related to the eventual presence of additional minor carotenoids, such as xanthophylls [42]: this aspect will be analyzed in a future work. Fresh EVOO samples, rich of chlorophylls, were also analyzed, by using the new modified method, at different times from their production. The spectra could be well reproduced by using four pigments: β-carotene, lutein, chlorophyll a, and pheophytin a. In these samples the addition of chlorophyll b and pheophytin b among pigments did not vary significantly the spectral deconvolution. This aspect can be considered a limitation of the present method with respect to HPLC standard method of pigments’ quantification. However, in contrast with common chromatographic methods, the proposed spectroscopic approach reduced significantly the time of analysis to about 1–2 min. In the present paper, the newly implemented spectroscopic method was used to determine the concentration of pigments of several fresh EVOOs during optimal storage conditions, namely T = 4 °C, green bottles in the dark. The kinetic of pigments’ degradation was finally investigated by using appropriate models [26,58,59,60]. These further experiments used to test and validate the spectroscopic method allowed us to determine the kinetic constants of both chlorophyll a and pheophytin a degradation in EVOOs stored in optimal conditions, confirming previous studies of kinetics of pigments in EVOOs [26,58]. The ability of the implemented spectroscopic method to evaluate the concentration of both chlorophylls and pheophytins is particularly interesting if we consider that in most of the cases actual spectroscopic methods [28,29,50] allow one to quantify only the total of chlorophylls’ derivatives and not the single components. This spectroscopic method is relatively easy to use and it combines the advantage of UV-vis spectroscopy (low cost and fast measurements) with the mathematical deconvolution approach to determine the pigments’ concentrations giving rise to a fast, cheap and useful tool for extra virgin olive oil characterization and quality assessment. 

## Figures and Tables

**Table 1:** EVOO samples used in the present work. Cultivar (with prevalence of one or two cultivars), or blend of different cultivars are indicated. Labels of each sample, geographic origin, day of oil production, and temperature of storage are also reported.

Label	Cultivar	Geographic Origin	Year of Oil Production (Day/Month/Year)	Storage Temperature
A	Leccino, Frantoio	Italy, Tuscany	28 October 2017	4 °C
B	Frantoio, Leccino	Italy, Tuscany	6 November 2017	4 °C
C	Coratina, Ogliarola	Italy, Apulia	15 November 2017	4 °C
D	Blend ^1^	Italy, Tuscany	9 November 2017	4 °C
E	Blend ^1^	Italy, Tuscany	23 October 2017	4 °C
F	Blend ^1^	Italy, Tuscany	24 October 2017	4 °C
G	Blend ^2^	Italy, Apulia	15 November 2017	4 °C
T1	Frantoio	Italy, Tuscany	11 November 2016	22 °C
T2	Moraiolo	Italy, Tuscany	9 November 2016	22 °C
T3	Leccino	Italy, Tuscany	12 November 2016	22 °C
T4	Pendolino	Italy, Tuscany	15 November 2016	22 °C

^1^ EVOO samples with a prevalence of Moraiolo cultivar. ^2^ EVOO samples with a prevalence of *Cellina di Nardò* cultivar.

**Table 2:** Values of chlorophyll a and chlorophyll b (ppm) as obtained from the deconvolution method applied to the analysis of UV-vis absorption spectra of the test samples, prepared as reported in the text. The value of *R*^2^ is also shown.

Samples’ Label	Chlorophyll a	Chlorophyll b	*R* ^2^
CA_5	4.98 ± 0.02 ppm	0	0.992
CB_5	0	4.97 ± 0.02 ppm	0.997
CA_15	15.03 ± 0.03 ppm	0	0.991
CB_15	0	14.96 ± 0.03 ppm	0.992
CAB_5_5	4.96 ± 0.02 ppm	4.98 ± 0.01 ppm	0.993
CAB_15_15	14.97 ± 0.04 ppm	15.02 ± 0.03 ppm	0.994

**Table 3:** Values of concentration (expressed in ppm) of four main pigments in fresh EVOOs (i.e., chlorophyll a, pheophytin a, β-carotene and lutein) as obtained from the deconvolution analysis of UV-vis absorption spectra the EVOO samples after about 1 month from their production. Other pigments, if present, are in smaller amount to produce significant improvement of the spectral analysis as reported in the text. Total amount of pigments is also reported. For each EVOO sample the concentration values obtained from the best fitting analysis are shown with the respective value of *R*^2^.

Label	Chlorophyll a	Pheophytin a	β-Carotene	Lutein	Total Pigments	*R* ^2^
A	7.8 ± 0.2 ppm	7.5 ± 0.1 ppm	7.1 ± 0.1 ppm	3.0 ± 0.1 ppm	25.4 ± 0.5 ppm	0.995
B	5.6 ± 0.2 ppm	4.3 ± 0.2 ppm	6.5 ± 0.1 ppm	1.7 ± 0.1 ppm	18.1 ± 0.6 ppm	0.996
C	9.8 ± 0.2 ppm	6.0 ± 0.1 ppm	3.3 ± 0.2 ppm	10.4 ± 0.2 ppm	29.5 ± 0.7 ppm	0.997
D	5.4 ± 0.2 ppm	4.6 ± 0.2 ppm	5.5 ± 0.1 ppm	2.7 ± 0.1 ppm	18.2 ± 0.6 ppm	0.996
E	6.1 ± 0.2 ppm	2.9 ± 0.1 ppm	4.4 ± 0.1 ppm	3.4 ± 0.1 ppm	16.8 ± 0.5 ppm	0.995
F	5.9 ± 0.1 ppm	3.2 ± 0.1 ppm	3.4 ± 0.1 ppm	5.1 ± 0.1 ppm	17.6 ± 0.4 ppm	0.995
G	6.9 ± 0.2 ppm	4.2 ± 0.1 ppm	3.5 ± 0.1 ppm	7.4 ± 0.1 ppm	22.0 ± 0.5 ppm	0.996

**Table 4:** Values of concentration (expressed in ppm) of four main pigments in ‘on-the-shelf’ EVOOs (i.e., pheophytin a, pheophytin b, β-carotene and lutein) as obtained from the deconvolution analysis of UV-vis absorption spectra the EVOO samples, after about 4 months from their production. Total amount of pigments is also reported. For each EVOO sample the concentration values obtained from the best fitting analysis are shown with the respective value of *R*^2^.

Label	Pheophytin b	Pheophytin a	β-Carotene	Lutein	Total Pigments	*R* ^2^
T1	1.3 ± 0.5 ppm	16.0 ± 0.3 ppm	1.6 ± 0.1 ppm	8.0 ± 0.1 ppm	26 ± 1 ppm	0.997
T2	2.5 ± 0.2 ppm	30.1 ± 0.2 ppm	3.2 ± 0.1 ppm	10.3 ± 0.1 ppm	46.1 ± 0.6 ppm	0.998
T3	1.3 ± 0.2 ppm	10.9 ± 0.1 ppm	1.4 ± 0.2 ppm	5.7 ± 0.2 ppm	19.4 ± 0.7 ppm	0.997
T4	0.7 ± 0.2 ppm	9.5 ± 0.2 ppm	1.5 ± 0.1 ppm	6.5 ± 0.1 ppm	18.2 ± 0.6 ppm	0.998

**Table 5:** Initial concentration, [*C*]_0_ (ppm), of lutein in the four monocultivar not-fresh EVOO samples; kinetic constant, *k* (d^−1^), absolute value of *P*_0_, obtained analyzing the trends of concentration of lutein during time. ‘d’ stands for ‘day’.

	[C]_0_ (ppm)	k (d^−1^)	|P_0_| (ppm/d)
T1 sample (*Frantoio*)	9.875	0.001	0.0099
T2 sample (*Moraiolo*)	14.783	0.002	0.0296
T3 sample (*Leccino*)	7.125	0.001	0.0071
T4 sample (*Pendolino*)	7.897	0.001	0.0079

**Table 6:** Initial concentration of chlorophyll a and pheophytin a, [*C_a*]_0_ (ppm) and [*Ph_a*]_0_ (ppm), and two kinetic constants, *k*_1_ (days^−1^) and *k*_2_ (days^−1^), obtained from the fitting of experimental data reported in Figure 11 by using Equations (11) and (12) of the selected fresh EVOO samples (label A, C, D, and E).

	Sample A	Sample C	Sample D	Sample E
[*C_a*]_0_ (ppm)	14.4	11.4	6.8	9.0
*k*_1_ (days^−1^)	0.01	0.004	0.009	0.008
[*Ph_a*]_0_ (ppm)	1.2	4.5	2.0	0.0
*k*_2_ (days^−1^)	0.001	0.0008	0.0011	0.0009

## Data Availability

Not applicable.

## Supplementary Materials

The following are available online at https://www.mdpi.com/article/10.3390/foods10081891/s1, Figure S1: Evolution of the near-UV visible spectrum (ABS versus nm) of the sample CA_18 under the effect of light exposure. Spectra are recorded at different times (t) in expressed in minutes, Figure S2: Trend of the concentration of chlorophyll a as obtained from the deconvolution of the spectra at different times (minutes) of the sample CA_18 under the effect of light exposure, Figure S3: Evolution of the near-UV visible spectrum of the sample CA_18 stored in the dark (green glass bottles) during time. Optical path was of 1 cm. Spectra are recorded during time (t) in expressed in days (d). Triolein spectrum is also reported as spectral base-line, Table S1: Concentrations of chlorophyll a (C a) and pheophytin a (Ph a), expressed in ppm, of the CA_20.5 sample, obtained from the deconvolution of the experimental spectra obtained at different time during a process of ‘accelerated’ acidification, Table S2: Concentrations of chlorophyll b (C b) and pheophytin b (Ph b), expressed in ppm, of the CB_21.1 sample, obtained from the deconvolution of the experimental spectra obtained at different time during a process of ‘accelerated’ acidification, Table S3: Concentrations of pigments (b-carotene, lutein, pheophytin a and pheophytin b), expressed in ppm, of the ‘on-the-shelf’ samples (T1, T2, T3, T4), obtained from the deconvolution of the experimental spectra obtained at different time during storage. Time in expressed in days after oil production.
