# Supplementary material for: Study of the Evolution of Pigments from Freshly Pressed to ‘On-the-Shelf’ Extra-Virgin Olive Oils by Means of Near-UV Visible Spectroscopy
Source: Foods. 2021 Aug 15;10(8):1891. doi: 10.3390/foods10081891 (PMC8394633; doi:10.3390/foods10081891)
Supplement: Supplementary file 1 [file foods-10-01891-s001.zip › foods-1334529-supplementary.pdf]

Supporting Information

Study of the Evolution of Pigments from Freshly Pressed to 'On-the-Shelf' Extra-Virgin Olive Oils by Means of Near-UV Visible Spectroscopy

Eleonora Borello [1,2,†], Daniele Roncucci [1,3,4,†] and Valentina Domenici [1,*]

[1] Dipartimento di Chimica e Chimica Industriale, Università di Pisa, via Moruzzi 13, 56124 Pisa, Italy;
bore.89.ele@gmail.com (E.B.); daniele.roncucci@empa.ch (D.R.)
[2] Acque Industriali S.R.L., Via Bellatalla 1, Ospedaletto, 56121 Pisa, Italy
[3] Advanced Fibers, Empa Swiss Federal Laboratories for Materials Science and Technology, Lerchenfeldstrasse 5, 9014 St. Gallen, Switzerland
[4] Lab für Anorganische Chemie, ETH Zürich, HCI H 131 Vladimir-Prelog-Weg 1-5/10, 8093 Zürich, Switzerland
* Correspondence: valentina.domenici@unipi.it; Tel.: +39-(050)-221-9215 (ext. 267)
† Former students at the Dipartimento di Chimica e Chimica Industriale, Università di Pisa.

Section S1. *Effect of light exposure and different storage conditions on chlorophylls by near-UV visible spectroscopy (test samples).*

As an example of test, in Figure S1, the spectra recorded for the sample CA_18, stored in a transparent bottle at T=22°C, at different times are reported.

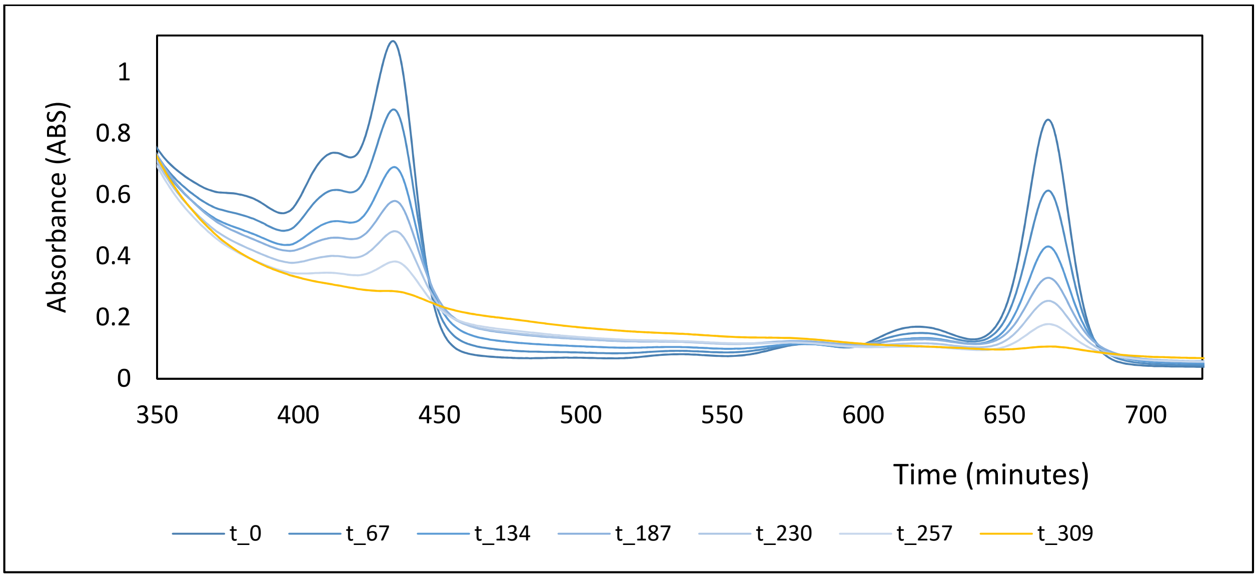

Figure S1. Evolution of the near-UV visible spectrum (ABS versus nm) of the sample CA_18 under the effect of light exposure. Spectra are recorded at different times (t) in expressed in minutes.

The sample consists of chlorophyll a (18 ppm) diluted in refined oil. The sample was exposed to sun light and the spectra were recorded until chlorophyll a was not detectable. Under light exposure, the degradation of chlorophylls is very fast and the sample becomes not colored.
The analysis of the spectra by using the deconvolution model gives rise to different concentrations of chlorophyll a versus time as reported in Figure S2.

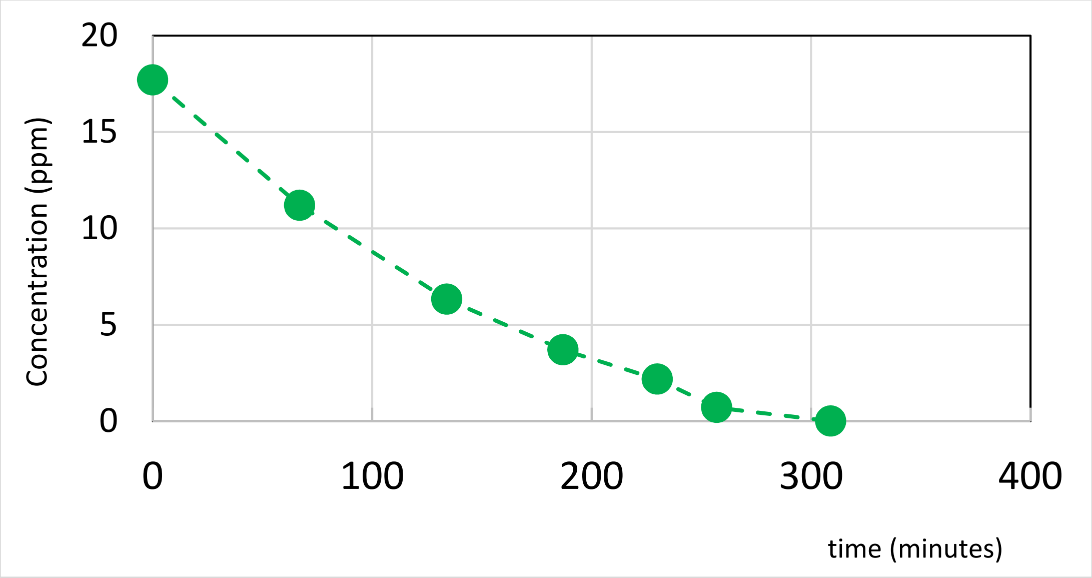

Figure S2. Trend of the concentration of chlorophyll a as obtained from the deconvolution of the spectra at different times (minutes) of the sample CA_18 under the effect of light exposure.

The deconvolution of the spectra was good in all cases, with $R^2 > 0.994$. The eventual amount of pheophytin a obtained from the fitting was less than 5% of the total pigments' content, meaning that the effect of light was to completely destroy the chromophore unit typical of chlorophylls' derivatives. Similar experiments were done by using green glass bottles in the dark (see Figure S3 with a sample containing chlorophyll a). Analogous experiments were performed with samples containing chlorophyll b or both pigments.

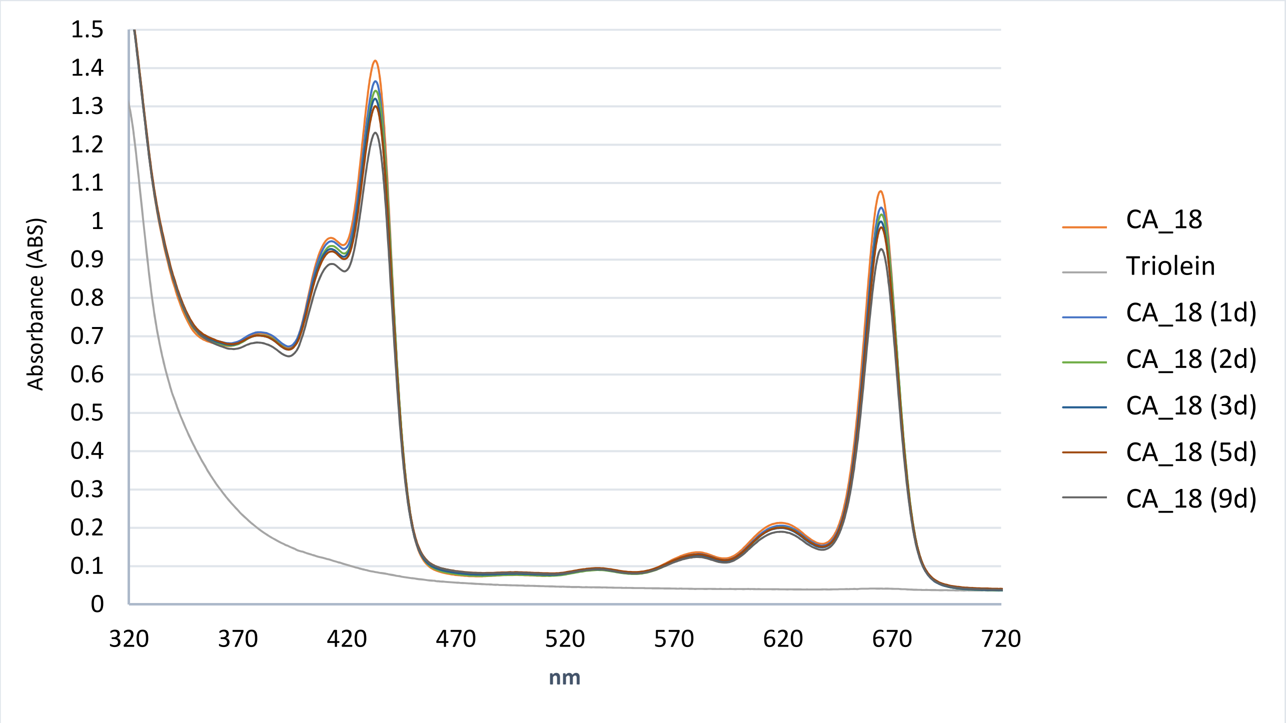

Figure S3. Evolution of the near-UV visible spectrum of the sample CA_18 stored in the dark (green glass bottles) during time. Optical path was of 1 cm. Spectra are recorded during time (t) in expressed in days (d). Triolein spectrum is also reported as spectral base-line.

Section S2. *Evolution of pigments during time in test samples.*

Data obtained from the deconvolution of the spectra reported in Figure 4 of sample CA_20.5, during two acidification steps:

Table S1. Concentrations of chlorophyll a (C a) and pheophytin a (Ph a), expressed in ppm, of the CA_20.5 sample, obtained from the deconvolution of the experimental spectra obtained at different time during a process of 'accelerated' acidification.

Time (')	C a (ppm)	Ph a (ppm)
0	20.9 ± 0.1	n.d
5	20.9 ± 0.1	n.d
9	20.7 ± 0.1	n.d
13	20.2 ± 0.1	n.d
17	20.3 ± 0.1	n.d
24	19.8 ± 0.1	n.d
31	19.8 ± 0.1	n.d
36	19.7 ± 0.1	~loq *
45	19.6 ± 0.1	~loq *
50	19.4 ± 0.1	0.2 ± 0.1
55	19.4 ± 0.1	0.3 ± 0.1
64	19.2 ± 0.1	0.5 ± 0.1
68	18.9 ± 0.1	0.7 ± 0.1
72	18.6 ± 0.1	1.0 ± 0.1
77	18.1 ± 0.1	1.5 ± 0.1
82	18.0 ± 0.1	1.6 ± 0.1
91	17.7 ± 0.1	1.9 ± 0.1
99	17.5 ± 0.1	2.1 ± 0.1
106	17.2 ± 0.1	2.4 ± 0.1
110	17.0 ± 0.1	2.6 ± 0.1
116	13.6 ± 0.1	5.8 ± 0.1
120	11.6 ± 0.1	7.8 ± 0.1
125	9.9 ± 0.1	9.4 ± 0.1
130	8.4 ± 0.1	10.9 ± 0.1
134	7.1 ± 0.1	12.1 ± 0.1
138	6.0 ± 0.1	13.3 ± 0.1
142	5.0 ± 0.1	14.2 ± 0.1
144	4.3 ± 0.1	14.9 ± 0.1
150	3.6 ± 0.1	15.6 ± 0.1
154	3.0 ± 0.1	16.2 ± 0.1
158	2.5 ± 0.1	16.7 ± 0.1
162	2.0 ± 0.1	17.1 ± 0.1

* loq is the limit of quantification that it was determined on the new model is 0.1.

Table S2. Concentrations of chlorophyll b (C b) and pheophytin b (Ph b), expressed in ppm, of the CB_21.1 sample, obtained from the deconvolution of the experimental spectra obtained at different time during a process of 'accelerated' acidification.

Time (')	C b (ppm)	Ph b (ppm)
0	21.2 ± 0.1	n.d
6	20.1 ± 0.1	n.d
10	20.2 ± 0.1	n.d
15	20.1 ± 0.1	n.d
19	20.0 ± 0.1	n.d
23	20.1 ± 0.1	n.d
27	20.1 ± 0.1	n.d
32	20.0 ± 0.1	n.d
40	20.0 ± 0.1	n.d
46	19.4 ± 0.1	~loq *
51	19.0 ± 0.1	~loq *
63	18.6 ± 0.1	0.4 ± 0.1
67	18.4 ± 0.1	0.5 ± 0.1
77	16.8 ± 0.1	1.2 ± 0.1
81	16.3 ± 0.1	1.6 ± 0.1
85	15.8 ± 0.1	2.1 ± 0.1
89	15.3 ± 0.1	2.5 ± 0.1
93	14.7 ± 0.1	2.9 ± 0.1
97	14.3 ± 0.1	3.3 ± 0.1
102	13.7 ± 0.1	3.8 ± 0.1
106	13.3 ± 0.1	4.1 ± 0.1
112	12.8 ± 0.1	4.5 ± 0.1
118	12.0 ± 0.1	5.1 ± 0.1
121	11.6 ± 0.1	5.4 ± 0.1
130	10.8 ± 0.1	6.1 ± 0.1
136	10.3 ± 0.1	6.5 ± 0.1
140	10.0 ± 0.1	6.7 ± 0.1
144	9.7 ± 0.1	7.0 ± 0.1
149	9.2 ± 0.1	7.3 ± 0.1
153	8.9 ± 0.1	7.5 ± 0.1

* loq is the limit of quantification that it was determined on the new model is 0.1.

Section S3. *Evolution of pigments in EVOO samples.*

Table S3. Concentrations of pigments (β-carotene, lutein, pheophytin a and pheophytin b), expressed in ppm, of the 'on-the-shelf' samples (T1, T2, T3, T4), obtained from the deconvolution of the experimental spectra obtained at different time during storage. Time in expressed in days after oil production.

T1 Sample				
**Day after oil production**	**β-Carotene (ppm)**	**Pheophytin b (ppm)**	**Pheophytin a (ppm)**	**Lutein (ppm)**
83	2.7 ± 0.3	1.5 ± 0.1	17.1 ± 0.2	8.3 ± 0.2
128	1.9 ± 0.3	1.3 ± 0.1	16.1 ± 0.2	8.2 ± 0.2
176	1.5 ± 0.3	1.3 ± 0.1	16.0 ± 0.2	8.0 ± 0.2
320	1.6 ± 0.3	1.1 ± 0.1	15.7 ± 0.2	6.4 ± 0.2
380	2.3 ± 0.3	1.3 ± 0.1	16.5 ± 0.2	5.4 ± 0.2
791	1.7 ± 0.3	0.8 ± 0.1	15.7 ± 0.2	3.0 ± 0.2
T2 Sample				
**Day after Oil Production**	**β-Carotene (ppm)**	**Pheophytin b (ppm)**	**Pheophytin a (ppm)**	**Lutein (ppm)**
83	3.50 ± 0.02	2.538 ± 0.004	31.23 ± 0.09	11.90 ± 0.01
128	3.01 ± 0.02	2.439 ± 0.004	30.06 ± 0.09	11.16 ± 0.01
176	3.22 ± 0.02	2.519 ± 0.004	30.05 ± 0.09	10.26 ± 0.01
320	3.06 ± 0.02	2.071 ± 0.004	29.24 ± 0.09	7.89 ± 0.01
380	3.00 ± 0.02	1.899 ± 0.004	29.53 ± 0.09	7.04 ± 0.01
841	1.77 ± 0.02	1.136 ± 0.004	23.77 ± 0.09	2.49 ± 0.01
T3 Sample				
**Day after Oil Production**	**β-Carotene (ppm)**	**Pheophytin b (ppm)**	**Pheophytin a (ppm)**	**Lutein (ppm)**
83	1.3 ± 0.3	1.1 ± 0.1	11.2 ± 0.2	6.5 ± 0.2
128	1.5 ± 0.3	1.2 ± 0.1	10.6 ± 0.2	5.9 ± 0.2
176	1.4 ± 0.3	1.3 ± 0.1	10.9 ± 0.2	5.7 ± 0.2
320	1.3 ± 0.3	1.1 ± 0.1	10.5 ± 0.2	4.8 ± 0.2
380	1.8 ± 0.3	1.2 ± 0.1	10.9 ± 0.2	4.1 ± 0.2
791	1.4 ± 0.3	0.8 ± 0.1	10.5 ± 0.2	2.6 ± 0.2
T4 Sample				
**Day after Oil Production**	**β-Carotene (ppm)**	**Pheophytin b (ppm)**	**Pheophytin a (ppm)**	**Lutein (ppm)**
83	2.6 ± 0.1	0.81 ± 0.03	10.3 ± 0.1	6.6 ± 0.2
128	1.8 ± 0.1	0.57 ± 0.03	9.4 ± 0.1	6.7 ± 0.2
176	1.5 ± 0.1	0.68 ± 0.03	9.5 ± 0.1	6.5 ± 0.2
320	1.7 ± 0.1	0.61 ± 0.03	9.4 ± 0.1	5.3 ± 0.2
380	1.8 ± 0.1	0.46 ± 0.03	9.5 ± 0.1	4.9 ± 0.2
791	1.7 ± 0.1	0.43 ± 0.03	9.5 ± 0.1	2.7 ± 0.2
